# Case report of delayed treatment of Jael’s syndrome: knife blade retained in maxillofacial region for 2 years

**DOI:** 10.1093/jscr/rjag993

**Published:** 2026-11-04

**Authors:** Pin-Yang Chen, Yi-Chia Wu, Ming-Jer Tsai, Su-Shin Lee

**Affiliations:** Department of Surgery, School of Medicine, College of Medicine, Kaohsiung Medical University, 4F, International Academic Research Building, 100, Shih-Chuan 1st Road, Kaohsiung 80708, Taiwan; Department of General Medicine, Kaohsiung Medical University Hospital, 6th floor, No. 100, Tzyou 1st Rd, San-Min District, Kaohsiung 80708, Taiwan; Division of Plastic Surgery, Department of Surgery, Kaohsiung Medical University Hospital, 19th floor, No. 100, Tzyou 1st Rd, San-Min District, Kaohsiung 80708, Taiwan; Regenerative Medicine and Cell Therapy Research Center, Kaohsiung Medical University, 9th floor, International Academic Research Building, 100, Shih-Chuan 1st Rd, San-Min District, Kaohsiung, 80708, Taiwan; Department of Surgery, Faculty of Medicine, College of Medicine, Kaohsiung Medical University, 12th floor, No. 100, Tzyou 1st Rd, San-Min District, Kaohsiung 80708, Taiwan; Division of Plastic Surgery, Department of Surgery, Kaohsiung Medical University Hospital, 19th floor, No. 100, Tzyou 1st Rd, San-Min District, Kaohsiung 80708, Taiwan; Division of Plastic Surgery, Department of Surgery, Kaohsiung Medical University Hospital, 19th floor, No. 100, Tzyou 1st Rd, San-Min District, Kaohsiung 80708, Taiwan; Regenerative Medicine and Cell Therapy Research Center, Kaohsiung Medical University, 9th floor, International Academic Research Building, 100, Shih-Chuan 1st Rd, San-Min District, Kaohsiung, 80708, Taiwan; Department of Surgery, Faculty of Medicine, College of Medicine, Kaohsiung Medical University, 12th floor, No. 100, Tzyou 1st Rd, San-Min District, Kaohsiung 80708, Taiwan

**Keywords:** Jael’s syndrome, penetrating craniofacial injury, penetrating facial injury, retained knife blade

## Abstract

Reports on Jael’s syndrome, characterized by intentionally inflicted knife wounds to the craniofacial region, are scarce. A 41-year-old man presented with a retained knife blade in the maxillofacial region 2 years after an assault. The prolonged delay in treatment resulted from his incarceration after the injury. Clinical evaluation revealed a stab wound penetrating from the right inferior orbital rim through the nasal septum into in the left maxillary sinus. Exploratory antrostomy and dacryocystorhinostomy were performed for knife blade removal with an uneventful postoperative course. This study reviewed the existing literature and examined strategies for addressing retained knife blades in the craniofacial area. Most cases involving acute knife wounds underwent urgent surgical intervention. This case underscores the importance of a comprehensive assessment for retained blades in deep stab wounds. Despite a 2-year delay in treatment, simple extraction performed in this case had no adverse effects on the outcome during 9 months of follow-up.

## Introduction

The first case of Jael’s syndrome was reported by Jefferson in 1968, in which a penetrating craniofacial injury was caused by a tent peg in an adolescent boy [1]. Harris *et al*. defined Jael’s syndrome as intentionally knife-inflicted wounds to the craniofacial region [2].

Stab wounds often result from assaults or industrial accidents. Reports of penetrating knife injuries to the face are rare. This rarity is attributed to the fact that individuals instinctively use their hands to shield their faces during self-defense [3]. Such injuries pose considerable challenges for physicians. A blade’s size and location may render the blade difficult to access. Moreover, close proximity to vital structures can lead to complications such as airway obstruction and inflammatory responses in surrounding tissues.

In injuries involving retained foreign bodies, a thorough initial assessment and multidisciplinary approach are essential. For penetrating wounds involving retained metallic foreign bodies, computed tomography (CT) in multiple planes can localize the blade accurately. In cases of severe hemorrhage, angiography is required. In the emergency room, the trauma team’s priority is to secure a clear airway, followed by stabilizing the patient’s hemodynamic status. Subsequently, ophthalmologic, vascular, and neurologic evaluations should be performed [2–6]. In this report, we present our experience with the delayed treatment of a retained maxillofacial foreign body, which involved no vascular and ophthalmological compromise.

## Case report

A 41-year-old man presented to Kaohsiung Medical University Hospital, Kaohsiung, Taiwan, with a 2-year history of an untreated knife assault. The patient, who was arrested shortly after being injured, was incarcerated without receiving medical intervention. Over the preceding 5 months, the patient reported experiencing trismus, rhinorrhea, and right-sided tearing. The patient was conscious and fully oriented during the examination. A four-view X-ray of the patient’s skull and a CT scan revealed an 8-cm knife penetrating from the right inferior orbital rim, traversing the nasal septum, and terminating in the left maxillary sinus (Figs 1 and 2). Due to the absence of active bleeding, angiography was deemed unnecessary. Nasal endoscopy confirmed the presence of part of the knife within the nasal cavity (Fig. 3). On the basis of these findings, the patient was given a diagnosis of Jael’s syndrome and right nasolacrimal duct impairment.

**Figure 1:**
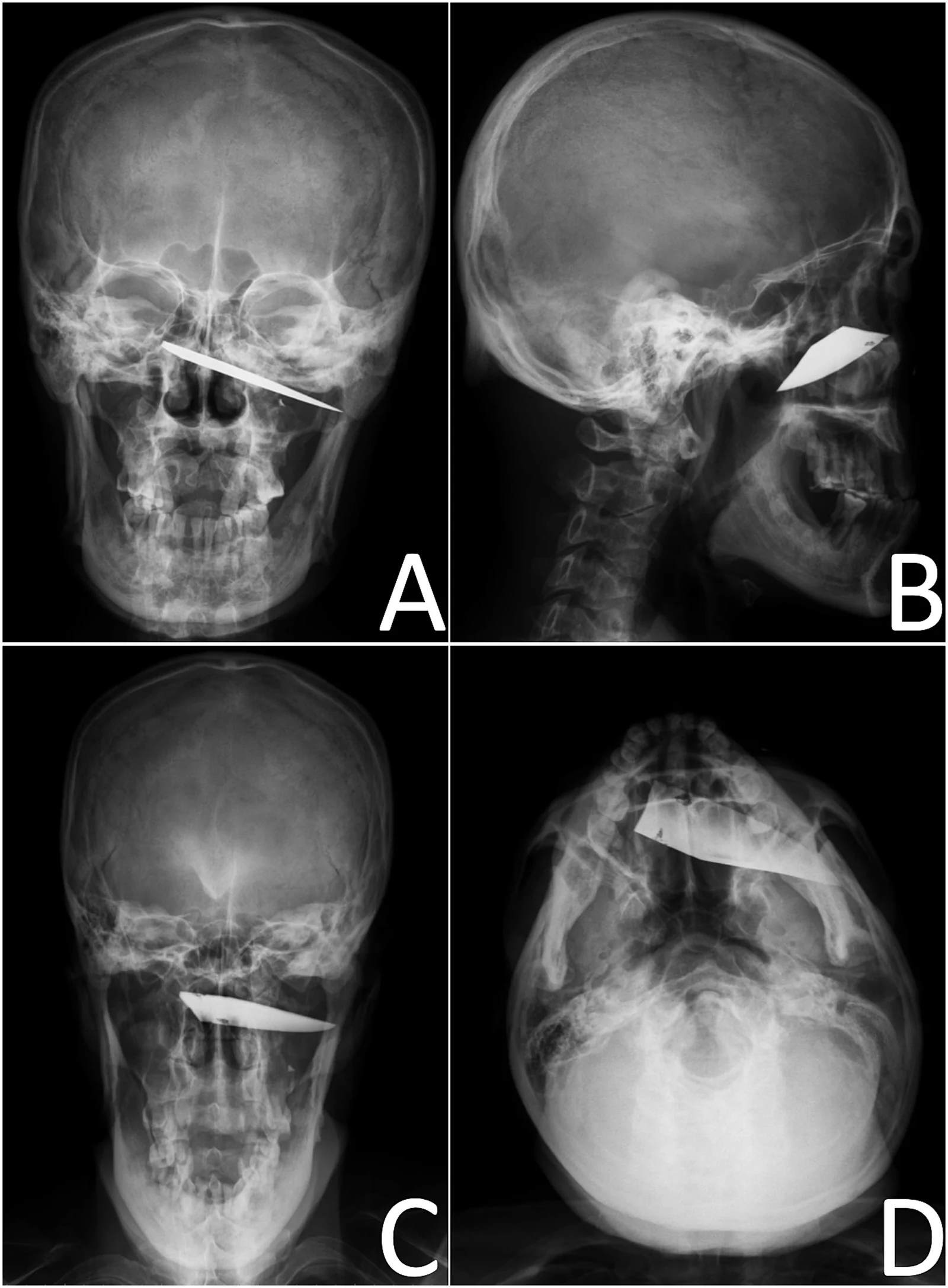
Knife (8 cm) penetrating from right inferior orbital rim, extending through nasal septum, and terminating in left maxillary sinus; (A) posterior–anterior view; (B) lateral view; (C) Towne view; (D) submental view.

**Figure 2:**
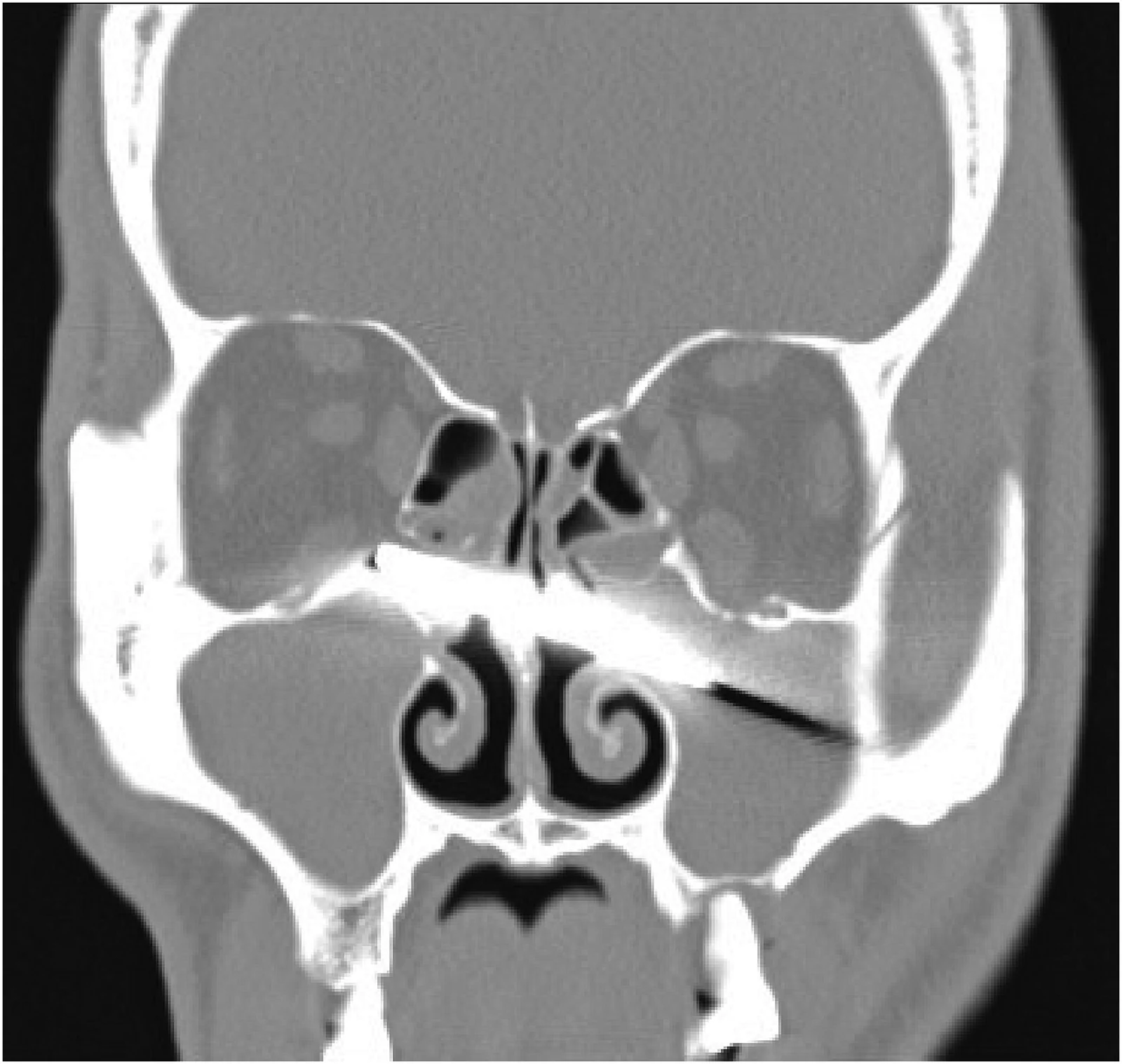
Preoperative coronal CT scan.

**Figure 3:**
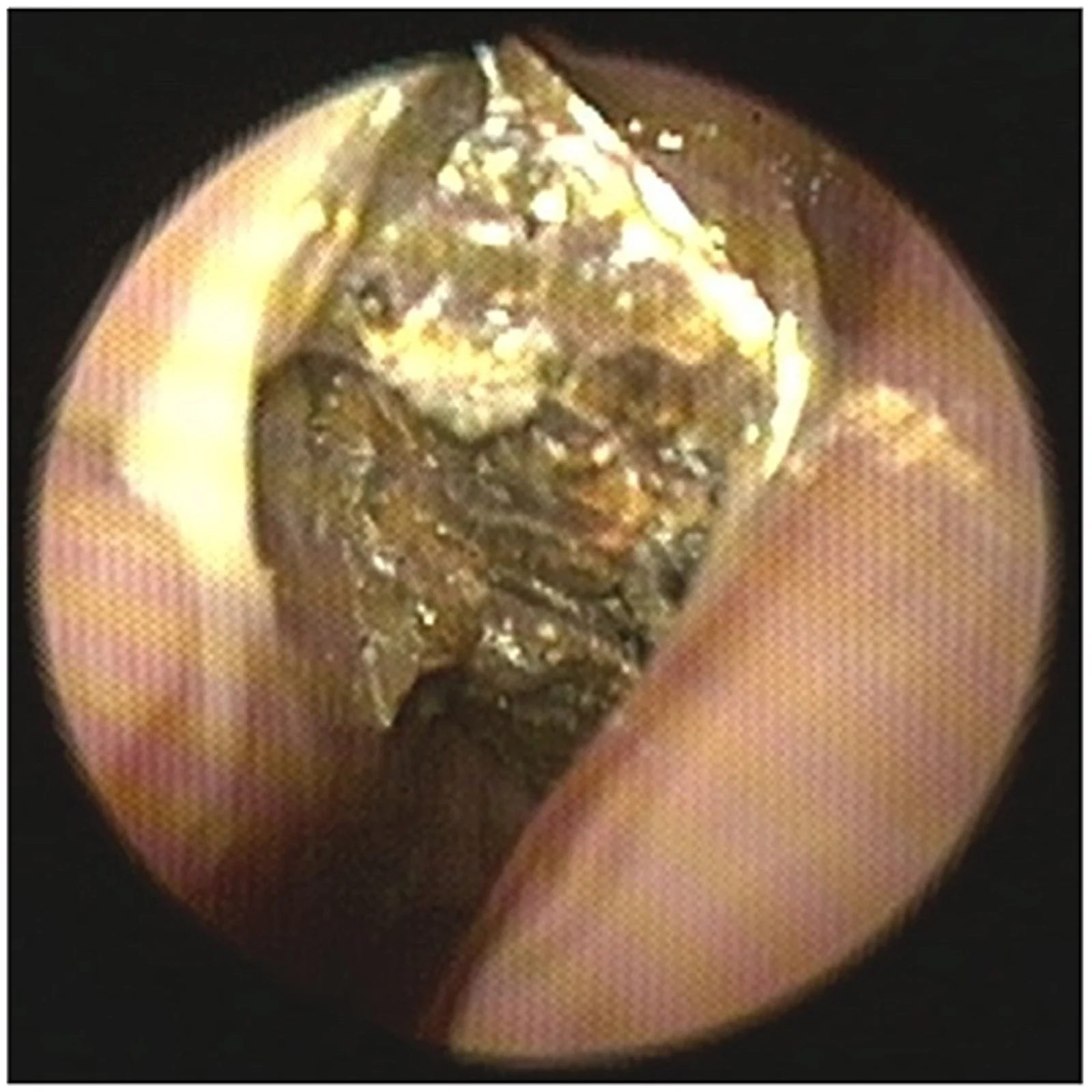
Portion of knife visible in nasal cavity during nasal endoscopy.

Surgical exploration was performed under general anesthesia. An exploratory antrostomy was conducted on the right maxillary sinus, and the 8-cm blade was removed along the insertion pathway with the aid of an endoscope (Fig. 4). A dacryocystorhinostomy of the right nasolacrimal duct was performed to address right-sided tearing and reconstruct the nasolacrimal system. The postoperative course was uneventful, and the patient was discharged after 3 days without immediate complications. During ~9 months of follow-up, no recurrent symptoms or late complications were documented.

**Figure 4:**
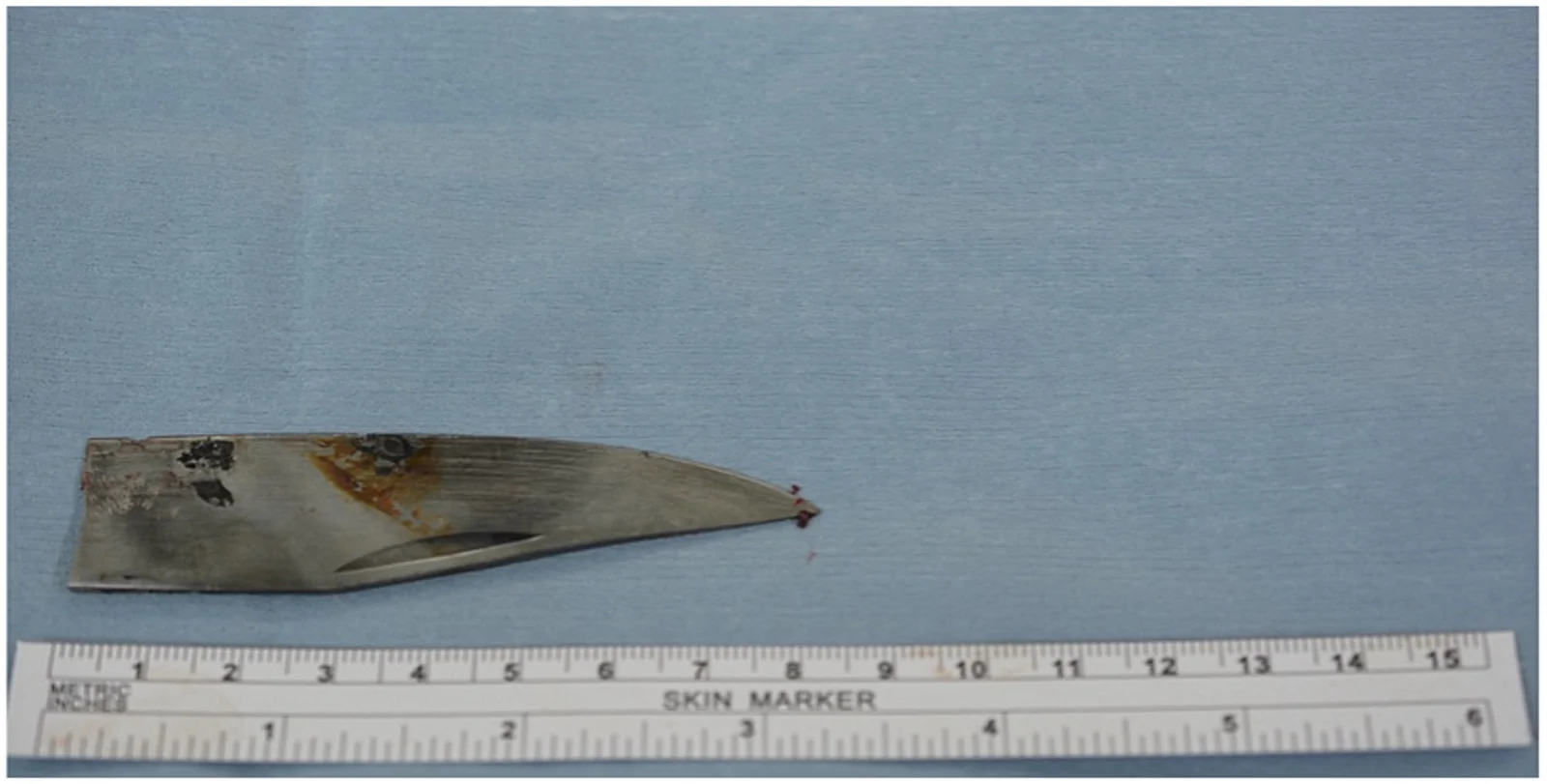
Removal of 8-cm retained knife blade.

## Discussion

An English literature review across PubMed was conducted to better understand the management and characteristics of Jael’s syndrome and retained knife blades using the following search terms: (Jael’s syndrome OR Jael syndrome) OR ((Retained knife blade) AND (Penetrating facial injury OR Penetrating craniofacial injury)). Cases that did not involve a knife-blade injury to the craniofacial region were excluded. Ten articles were selected, yielding a total of 17 patients for review. All reported cases involved male patients. Urgent surgical intervention was defined as definitive knife removal or surgical treatment during the initial hospitalization for the injury, irrespective of the exact injury-to-presentation interval. Delayed surgical intervention was defined when a treatment delay was explicitly documented in the original report. Among the 17 patients, 14 underwent urgent surgical intervention and 3 underwent delayed surgical intervention, with documented delays of ~1 week, 1.5 months, and 2 months, respectively (Supplementary Table S1). The majority of cases in the review highlighted the importance of immediate imaging and intervention in optimizing patient outcomes. In the present case, the patient’s complaint was overlooked at the local hospital due to the absence of active bleeding or airway compromise. This case represents an exceptionally rare scenario in clinical practice where a major delay of 2 years in formal imaging and treatment did not adversely affect outcomes. Van Lierop *et al*. reported a similar case involving a 2-month delay in the treatment of a stab injury in the left eye, with no further imaging performed after initial wound cleaning [4]. These cases emphasize the importance of maintaining a high level of suspicion in patients presenting with deep stab wounds, where the possibility of a retained knife blade cannot be excluded. A thorough evaluation of patient history is essential to confirm the presence of a retained blade, and a comprehensive head examination should include radiographs, cranial nerve assessments, and a nasoscopy for suspected pharyngeal or nasal injuries.

To accurately determine the location of a foreign body and its relationship with crucial anatomical structures, conventional X-rays or CT scans were performed in all reviewed articles to minimize the risk of false-negative findings [2–10]. In the present case, a four-view X-ray of the skull was performed initially to confirm the blade’s pathway and length because a CT machine was not immediately available. Standard four-view radiographs provide adequate information for surgeons to promptly assess injury severity. Subsequently, a CT scan was performed to evaluate potential damage to surrounding vascular structures and soft tissue. In 3 of the 17 reviewed cases, CT angiography was required due to considerable arterial bleeding or because the retained knife blades were located near or were suspected to involve vital arteries. Angiography can enable surgeons to identify bleeding vessels and plan selective embolization or surgical ligation [4, 6, 8].

In 10 of the reviewed cases, simple knife removal along the pathway resulted in no notable sequelae [2–8]. Simple extraction can generally be attempted in the operating room when vascular or cranial injuries are ruled out. However, in cases involving penetrating cranial injuries, craniotomy and dural repair may be indicated for blade extraction [8–10]. Gluncic *et al*. reported a case where the knife tip was in close proximity to the left internal carotid artery, and endovascular balloon occlusion was used to minimize bleeding during blade manipulation [8]. The present case had no involvement of other vessels or cerebral structures, allowing for simple knife removal. Simultaneously, the injured inferior canaliculus was repaired with a silicone stent to improve the patient’s quality of life.

## Conclusion

This case highlights the importance of a comprehensive evaluation to identify retained blades in deep stab wounds. Effective management requires detailed patient history-taking, radiographic imaging, cranial nerve assessments, and nasoscope examination. In most cases, surgical removal of the knife along its insertion pathway is both safe and effective. Remarkably, despite a 2-year treatment delay, our patient experienced no adverse outcomes following the simple knife extraction over ~9 months of follow-up.

## Supplementary Material

Supplementary_material_rjag993
