# Supplementary material for: Case report of delayed treatment of Jael’s syndrome: knife blade retained in maxillofacial region for 2 years
Source: J Surg Case Rep. 2026 Nov 4;2026(11):rjag993. doi: 10.1093/jscr/rjag993 (PMC13731183; doi:10.1093/jscr/rjag993)
Supplement: Supplementary file 1 [file supplementary_material_rjag993.docx]

| **Reference**  ***Supplemental Table 1. Review of Jael’s syndrome Cases in the Literature*** | **Age/Sex** | **Initial management** | **Timing and classification of surgical intervention** | **Radiology** | **Entry point** | **Site of blade** | **Length of the retained blade** | **Procedure and treatment** | **Outcome** |
| --- | --- | --- | --- | --- | --- | --- | --- | --- | --- |
| Harris AM *et al.^[1]^* | 24/Male | X ray | Delayed surgical intervention; presented 1.5 months after injury and subsequently underwent surgical removal | X ray | Left maxilla | Left ramus area | Not described | Simple knife removal | No sequelae |
|  | 28/Male | X ray | Urgent surgical intervention; presented with the retained knife and underwent surgical removal | X ray | Right temporal area | Right lateral wall of the orbit, with the tip near the left nasal bridge | Not described | Simple knife removal | No sequelae |
|  | 24/Male | X ray | Urgent surgical intervention; presented with the retained knife and underwent surgical removal | X ray | Left inferior orbital rim | Left maxillary sinus | 12cm (according to Figure 6) | Simple knife removal | No sequelae |
|  | 34/Male | X ray | Urgent surgical intervention; presented with the retained knife and underwent surgical removal | X ray | Eye socket | Deep craniofacial structures | Not described | Simple knife removal | No sequelae |
| van Lierop *et al.^[2]^* | 35/Male | X ray and CT scan | Delayed surgical intervention; presented 2 months after injury and subsequently underwent surgical removal | X ray and CT scan | Left upper eyelid | Nasal septum and right maxillary sinus | 13cm | Simple knife removal with lateral canthotomy and inferior conjunctival incision | No sequelae |
|  | 37/Male | X ray and CT scan | Urgent surgical intervention; presented 4 h after injury and underwent surgical removal | X ray and CT scan | Left temporal bone | Petrous bone | Not described | Simple knife removal with mastoidectomy and interposition greater auricular nerve graft | No sequelae |
| Dominguete *et al.^[3]^* | 30/Male | Nimesulide twice a day | Urgent surgical intervention; presented 5 days after injury and underwent surgical removal | X ray | Left nostril | Bilateral nasal fossa and the right maxillary sinus | 12.5 cm | Simple knife removal | No sequelae |
| Yarandi *et al.^[4]^* | 21/male | CT scan | Urgent surgical intervention;  the knife was removed immediately at the scene by the offender, followed by surgical treatment | CT scan | Right orbital rim | Frontal lobe | Not described | Elevation of the bone fragments, debridement of the contused brain, dura repair and reconstruction of the rim | No sequelae |
|  | 14/Male | X ray, CT scan | Delayed surgical intervention; Presented after developing symptoms 1 week after injury and subsequently underwent surgical removal of the retained knife fragment | X ray, CT scan | Right posterior frontal region | Right posterior frontal region | Not described | Removal of retained knife fragment, debridement, and drainage of brain abscess | No sequelae |
|  | 30/Male | CT scan | Urgent surgical intervention; presented with the retained knife and underwent surgical removal | CT scan | Medial canthus of right eye | Medial orbital wall | Not described | Simple knife removal with lesser wing decompression and enucleation of eye | Loss of right eye before surgery |
|  | 24/Male | CT scan and CT angiography | Urgent surgical intervention; presented with the retained knife and underwent surgical removal | CT scan and CT angiogra-phy | Lateral canthus of the left eye | Cavernous sinus | Not described | Fronto-orbito-lateral craniotomy, lesser/greater sphenoid wing decompression and knife removal | Mild limitation in the eversion of the injured eye |
| Dai *et al.^[5]^* | 25/Male | X ray | Urgent surgical intervention; underwent surgical removal 84 h after injury | X ray, CT scan | Inferior orbital rim | Left medial orbital wall, ethmoid air cells, turbinates and right sphenoid bone | 10.8 cm | Simple knife removal | No sequelae |
| Voss *et al.^[6]^* | 38/Male | X ray | Urgent surgical intervention; presented with the retained knife and underwent surgical removal | X ray | Right supraorbital | Neurocranium | Not described | Craniotomy, dura repair, and knife removal | No sequelae |
| Azzi *et al.^[7]^* | 30/Male | Intubation, X ray and CT scan | Urgent surgical intervention; presented with the retained knife and underwent surgical removal | X ray and CT scan | Left orbit | Temporal lobe | 12.2cm | Left frontoparietal-occipital craniotomy, partial temporal lobectomy and knife extraction | Persistent left pupil dilation and right superior homonymous quadrantano-pia |
| Gluncic *et al.^[8]^* | 18/Male | Sedation, CT scan, X ray, CT angiography | Urgent surgical intervention; presented with the retained knife and underwent surgical removal | CT scan, X ray, CT angiogra-phy | Left orbital floor | Maxillary sinus along the skull base | Not described | Endovascular balloon occlusion and knife removal | No sequelae |
| Abraham *et al.^[9]^* | 31/Male | X ray and CT scan | Urgent surgical intervention; presented 16 h after injury and underwent surgical removal | X ray and CT scan | Frontal region | Left frontal bone to the medial wall of the left orbit | Not described | Simple knife removal | No sequelae |
| Alam *et al.^[10]^* | 22/Male | Sedation and CT scan | Urgent surgical intervention; admitted approximately 40 min after injury and underwent surgical removal on the second day of admission | CT scan and CT angiogra-phy | Left maxillary sinus | Medial pterygoid process and nasopharyngeal area | 9 cm | Simple knife removal | Left upper lip weakness, hypoesthesia of left infraorbital n. |

Urgent surgical intervention was defined as definitive knife removal or surgical treatment during the initial hospitalization for the injury, regardless of whether the exact injury-to-presentation interval was reported. Delayed surgical intervention was defined only when the original report explicitly documented a delay between injury and definitive treatment.

Reference

1.	Harris AM, Wood RE, Nortjé CJ, Grotepass F. Deliberately inflicted, penetrating injuries of the maxillofacial region (Jael's syndrome). Report of 4 cases. J Craniomaxillofac Surg. 1988; 16: 60–3.

2.	van Lierop AC, Raynham O, Basson O, Lubbe DE. Retained knife blades in the ear, nose and throat: three cases. J Laryngol Otol. 2009; 123: 351–5.

3.	Dominguete PR, Matos BF, Meyer TN, Oliveira LR. Jael syndrome: removal of a knife blade impacted in the maxillofacial region under local anaesthesia. BMJ Case Rep. 2013; 2013.

4.	Yarandi KK, Jelodar S, Khalatbari MR, Rasras S, Ilkhchi RB, Amirjamshidi A. Stab Wounds to the Head; Case Series, Review of Literature, and Proposed Management Algorithm. Asian J Neurosurg. 2018; 13: 754–9.

5.	Dai D, Meyer S, Kaltheuner LC, Plani F. On a knife-edge: clinical uncertainty with an extensive knife blade in situ in the craniofacial region. BMJ Case Rep. 2018; 2018.

6.	Voss JO, Thieme N, Doll C, Hartwig S, Adolphs N, Heiland M, et al. Penetrating Foreign Bodies in Head and Neck Trauma: A Surgical Challenge. Craniomaxillofac Trauma Reconstr. 2018; 11: 172–82.

7.	Azzi AJ, Saluja R, Mankowski P, Wakil S, Arthurs B, Lessard L. Transcranial Blade Injuries and Principles of Their Safe Extraction. J Craniofac Surg. 2019; 30: e228–e31.

8.	Gluncic V, Lukić A, Hanko E, Lynch J. Anesthetic Management of Jael Syndrome With Impacted Blade in Close Proximity to the Internal Carotid Artery: A Case Report. A A Pract. 2019; 12: 369–71.

9.	Abraham ZS, Lomnyack WP, Kimario OM, Kahinga AA. Jael's syndrome: Case report and literature review. Int J Surg Case Rep. 2021; 88: 106484.

10.	Alam M, Golkar M, Badkoobeh A, Baseri M. Jael's syndrome: Hemostatic management of an impacted knife in the facial site-A case report. Clin Case Rep. 2023; 11: e8163.
